# Novel Diagnosis and Treatment for Neurogenic Thoracic Outlet Syndrome

**DOI:** 10.7759/cureus.71434

**Published:** 2024-10-14

**Authors:** Leonid Tafler, Sonia Borkowski, Ghazal Javaid, David Gandolfo, David Kleyn

**Affiliations:** 1 Primary Care, Touro College of Osteopathic Medicine, New York, USA; 2 Family Medicine, Touro College of Osteopathic Medicine, New York, USA; 3 Family Medicine, New York Institute of Technology (NYIT), Old Westbury, USA

**Keywords:** neck pain, neurogenic thoracic outlet syndrome, neuropathy, osteopathic manipulation treatment, osteopathic manipulative medicine, osteopathic manipulative medicine (omm), physical therapy, thoracic outlet syndrome, tos

## Abstract

This article presents a unique diagnostic test for the neurogenic thoracic outlet syndrome (nTOS). nTOS is one of the most misdiagnosed and controversial medical problems; the diagnosis is clinical, and there are few specific diagnostic criteria for this condition. We would like to share this unique diagnostic modality, the Tafler test, with medical professionals. The Tafler test helps diagnose nTOS, differentiate it from cervical radiculopathy and carpal tunnel syndrome, and effectively tailor treatment for its symptoms. The following case series aims to describe several patients with nTOS who had failed previous treatment with surgery, physical therapy, and analgesics. The implementation of the Tafler test as a treatment modality in combination with osteopathic manipulative treatment (OMT) and physical therapeutic modalities led to significant improvements in treatment efficiency.

## Introduction

Thoracic outlet syndrome is an umbrella term representing a complex set of signs and symptoms due to various causes. First described in 1956 as a heterogenous group of symptoms, the common denominator is the compression of nerves or vascular structures as they pass through the thoracic inlet, traverse the shoulder girdle and axilla, and begin their descent into the arm [1]. Three passageways are of particular concern. One is the triangle formed by the anterior and medial scalene muscles and the first rib. The second passageway is between the clavicle and the first rib, and the third is along the border of the pectoralis minor near its attachment to the coracoid process [2]. Several mechanisms may be responsible for the symptoms of neurogenic thoracic outlet syndrome (nTOS), including traumatic injury, repetitive motions such as head and neck straining during computer work, cell phone use, physical exercise, anatomic variations, or malignancy [3].

Unfortunately, in many cases, this condition has been mistakenly attributed to a cervical pathology such as disc herniation, prompting medical professionals to choose medical massage and stretching as the main physiotherapeutic treatment, or refer for surgical intervention. Imaging modalities, including MRI, can be useful for diagnosing neuropathy secondary to internal or external compression, as well as electromyography (EMG) and nerve conduction studies. However, MRI cannot be used to rule out nTOS because of its low sensitivity to detect nerve compression secondary to muscle hypertrophy [4]. Additionally, although several studies have outlined and compared surgical methods for correcting nTOS, there has not been any comprehensive, systematic comparison of surgical and non-invasive interventions for the treatment of nTOS [5,6]. Thus, this article aims to present a diagnostic technique for nTOS, developed and applied for over 10 years. This method is useful for differentiating nTOS from cervical nerve impingement due to a herniated disc and carpal tunnel syndrome. Our article presents several cases of misdiagnosed or correctly identified nTOS treated with various modalities, many found to be ineffective. The patients subsequently presented to the osteopathic family practice where the novel diagnostic test was determined to be positive for nTOS. Applied in practice for over a decade and successfully treated more than 50 patients, this test can prevent misdiagnosis of nTOS and avoid unnecessary treatment that may aggravate symptoms. Manipulation under anesthesia incorporating the Tafler test with osteopathic modalities was found to be successful in resistant cases [7].

## Case presentation

Case A: three months of physical therapy and massage aggravating pain

A 56-year-old female began feeling severe right-sided neck pain radiating to her right arm and upper back. She reports that she had gone to the gym that day and the pain started after her workout. She was unable to move her arm without discomfort, causing a disturbance in her daily activities and sleep. Based on her clinical presentation, her primary care physician diagnosed her with cervical radiculopathy, and she was subsequently referred for physical therapy. The patient did not obtain any x-ray, MRI, or other imaging to confirm this diagnosis because her physician ruled it was not indicated following the accepted standard of care. She continued to receive treatment consisting of a massage, warm compresses, and electrical stimulation. The patient reported that after each visit, her pain got progressively worse and spread to her mid-back and right arm. She also developed numbness in her right hand; the application of a massage commonly used for inflamed muscles aggravated her symptoms. She started taking non-steroidal anti-inflammatory drugs (NSAIDs) to alleviate her discomfort. After three months of unsuccessful treatment, she presented to the osteopathic clinic, where the Tafler test was performed and determined to be positive for nTOS. The maneuver was maintained in a steady position for up to 60 seconds, providing sufficient time to ensure stretching of the affected tissues and leading to the release of the nerve. The patient was successfully treated using the Tafler test as a therapeutic modality in combination with osteopathic manipulative treatment (OMT) (myofascial release, muscle energy, and counterstrain technique). She received this treatment for two weeks, after which she no longer complained of pain.

Case B: six months intensive physical therapy with massage as the main therapeutic entity

**Figure 1:**
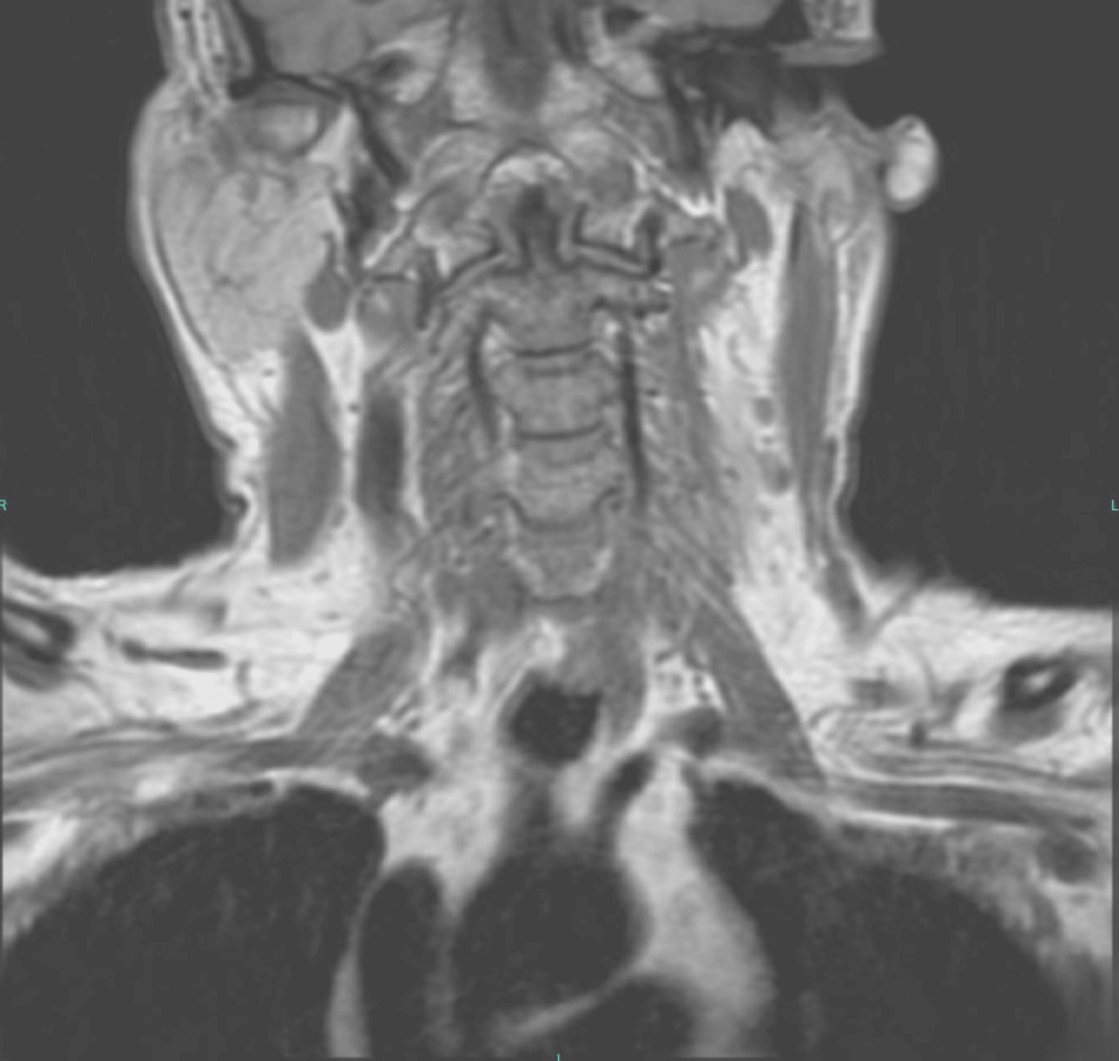
Case B: MRI of brachial plexus. There is no intrinsic mass or extrinsic compression of the left brachial plexus.

A 58-year-old male with a history of chronic back pain reported initial neck and interscapular pain. He explained that he was carrying two grocery bags up some stairs when he felt a sharp pain and had to instantly put his bags down. Over-the-counter pain medications provided little relief. He was referred to a chiropractor, and after three adjustments, he still felt no relief and was referred to an orthopedic surgeon. The surgeon diagnosed thoracic outlet syndrome (TOS) and ruled out other causes of nerve compression through obtaining an MRI (Figure 1). He offered surgery to treat the patient, which would involve the removal of the patient’s top rib to alleviate the pain. Before moving forward with the surgery, he encouraged the patient to try physical therapy. The patient received six treatment sessions consisting of stretching, exercises with resistance bands, and massage. He reported that his pain got worse and had spread to his left hand, specifically after the massage. He denied any numbness or tingling at the time. He was then referred to the osteopathic clinic, where the Tafler test was performed and found to be positive for nTOS. The patient was successfully treated using the Tafler test as a therapeutic modality (as described in Case A) in combination with OMT (myofascial release, muscle energy, and counterstrain technique).

Case C: two months of massage and scheduled cervical surgery

**Figure 2:**
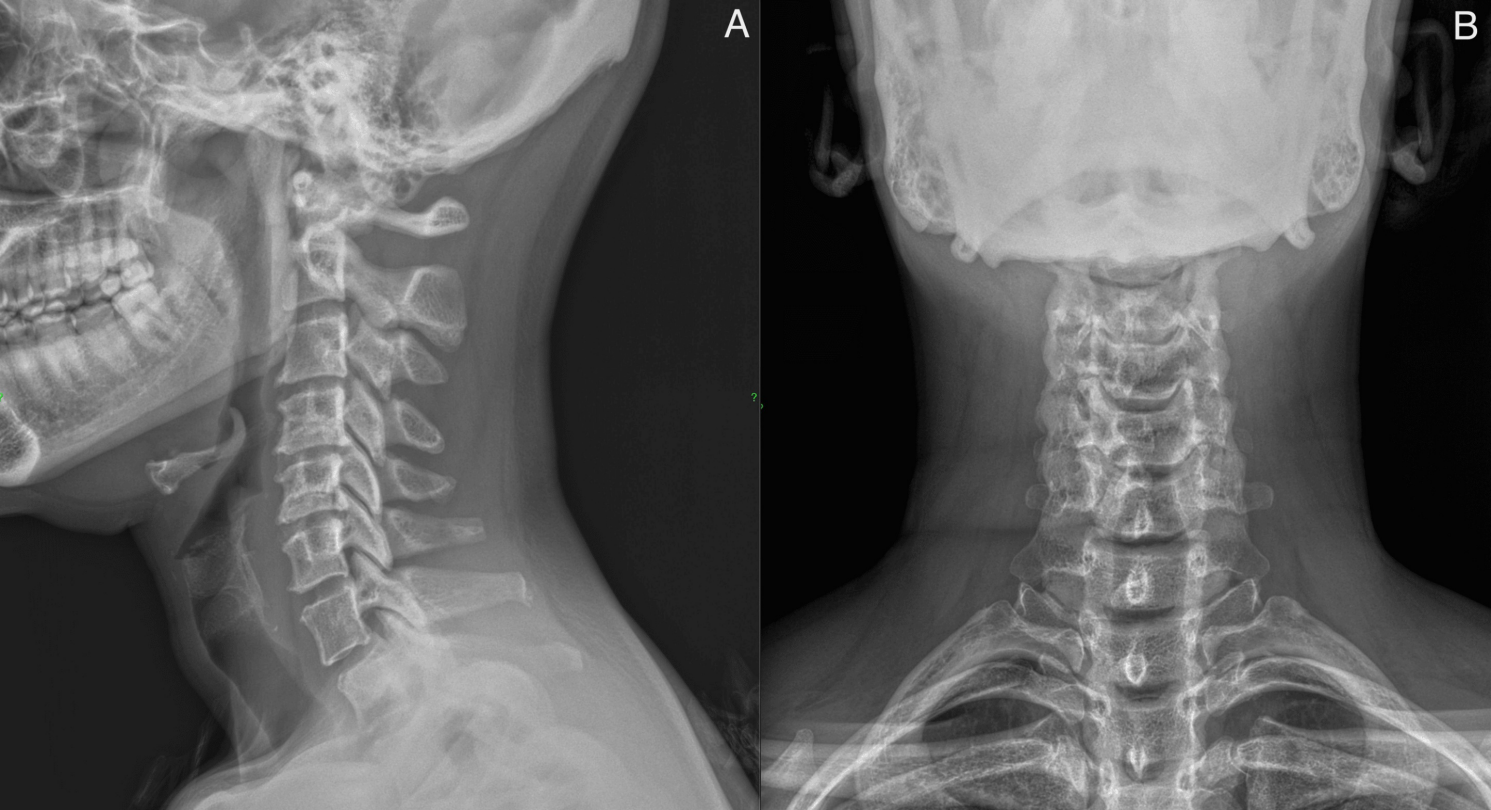
Case C: X-Ray cervical spine, lateral (A) and AP (B) views. There is mild reduction in disc space height at C4-5 and C5-6. Straightening of the cervical lordosis indicative of muscle spasm.

The following patient is a 53-year-old female who presented to the osteopathic family practice with complaints of worsening left-sided neck pain, stiffness, and intermittent paresthesia of the right arm and first and second digits after three months of physical therapy. Obtaining a detailed history, the patient stated that the neck pain began gradually about three months ago followed by paresthesia about one month later. She denied any trauma or sports injury but admitted to frequently carrying and walking with heavy shopping bags over her shoulder. Shortly following the onset of her symptoms, the patient sought physical therapy, which included electrical stimulation and massage of the cervical muscles. After about 18 visits over the two months following symptom onset, the patient reported no improvement and stated that the numbness and tingling in her digits intensified following each session. The patient was subsequently referred to an orthopedic surgeon for further management, who obtained an x-ray of her cervical spine (Figures 2A, 2B). The orthopedic surgeon recommended exploratory surgical intervention to correct possible cervical disc herniation and nerve impingement. Deciding to seek other opinions before proceeding with further imaging and the invasive procedure, the Tafler test was used to diagnose this patient’s nTOS and found to be positive. The patient was successfully treated using Tafler test as a therapeutic modality (as described in Case A) in combination with OMT (myofascial release, muscle energy, and counterstrain technique).

Case D: cervical surgery performed for disc herniation

**Figure 3:**
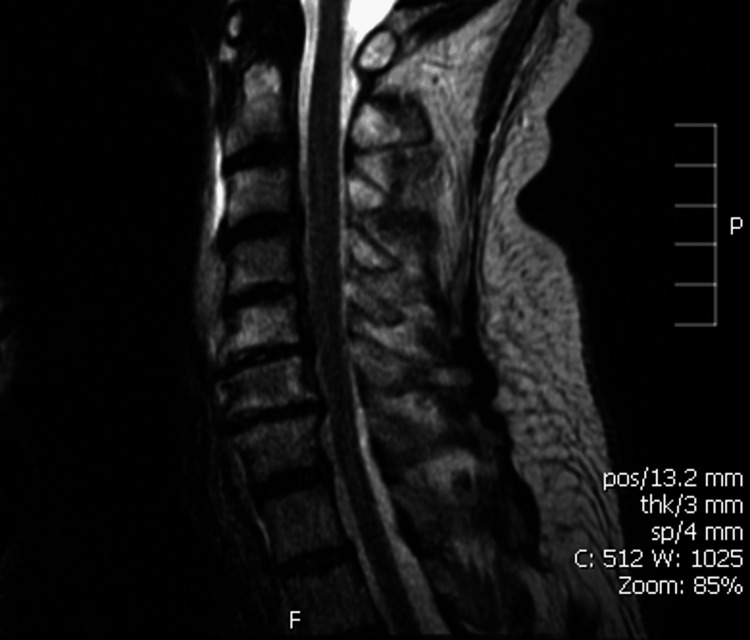
Case D: MRI before surgery. There is moderate to marked right C6 root compression. There is mild posterior osteophyte formation at C6-7 causing mild thecal sac deformity and moderate asymmetric left C7 root compression.

A 59-year-old male patient presented with complaints of weakness in his right arm following a skiing accident. In the two weeks after the accident, physical examination revealed notable asymmetry of the right shoulder girdle accompanied by significant atrophy of the deltoid, brachioradialis, and biceps muscles. Vascular assessment revealed no signs of compromise. However, the shoulder range of motion was severely restricted with flexion, abduction, and internal rotation registering at zero degrees, and extension at only five degrees. Neurologic testing including the Adson, Spurling, and Foramen compression tests returned negative results, helping to clinically rule out nerve root compression. Despite these findings, MRI revealed right-sided C6 nerve compression and some asymmetric compression of C7 (Figure 3).

**Figure 4:**
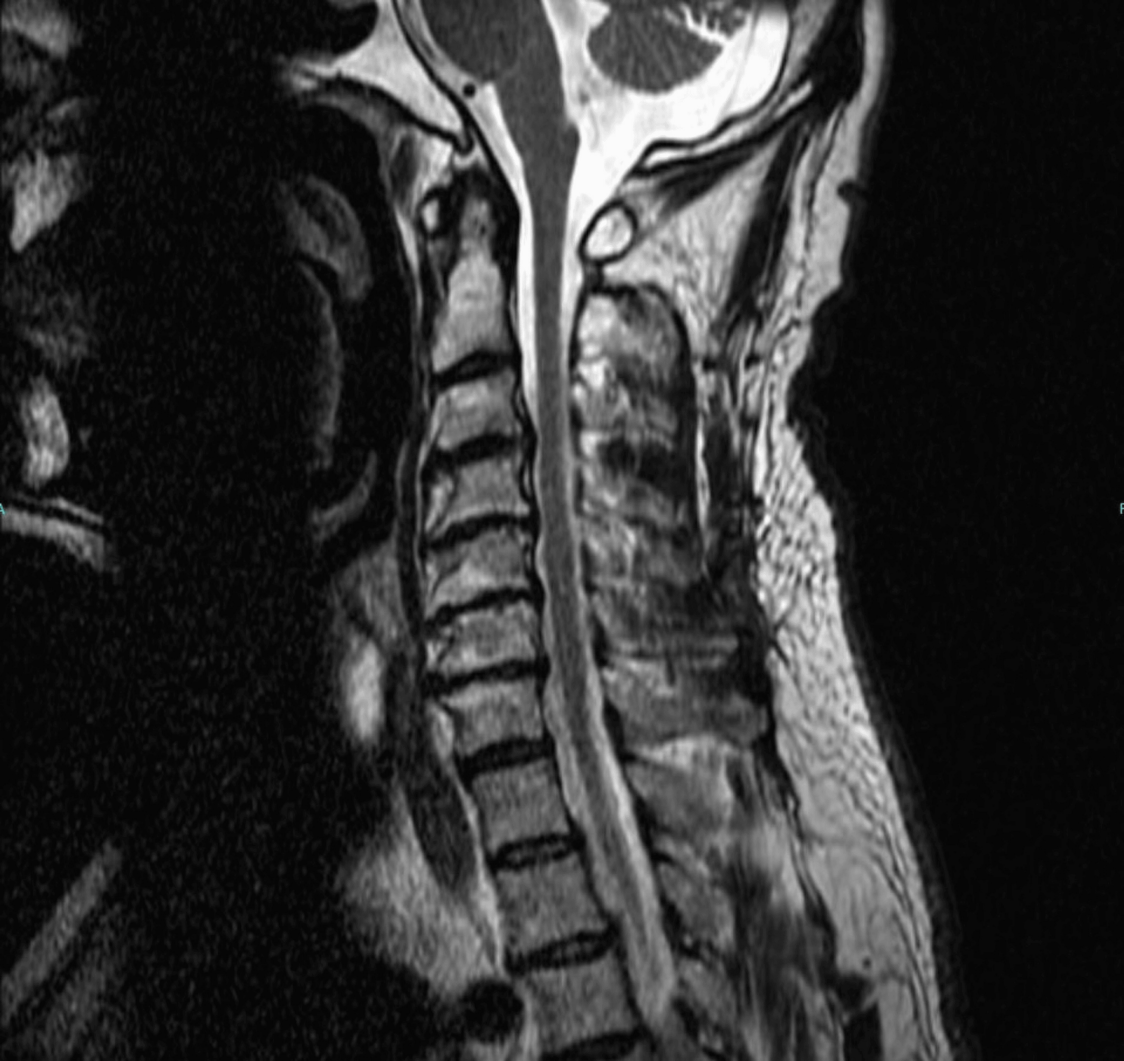
Case D: Post-surgical MRI. There is no evidence of spinal cord compression. There is mild nerve root impingement predominantly at C5-6 and C6-7 disc levels.

The patient saw a neurologist for further evaluation, who decided cervical radiculopathy and TOS were the two most likely differential diagnoses. Consulting with a neurosurgeon, the patient decided to proceed with surgical cervical intervention which unfortunately did not yield the desired outcome. Figure 4 depicts the patient’s post-surgery MRI taken while the patient’s symptoms were still present. Three months later, the patient sought care from an osteopathic physician, who performed the Tafler test and found it to be positive for nTOS. Osteopathic manipulation under anesthesia was applied, resulting in a remarkable improvement evidenced by the patient’s ability to flex his right elbow just 15 minutes post-procedure. Over five months, he experienced a substantial recovery, gaining back right shoulder muscle with progress continuing thereafter.

Case E: correctly diagnosed nTOS and multiple successful osteopathic treatments

**Figure 5:**
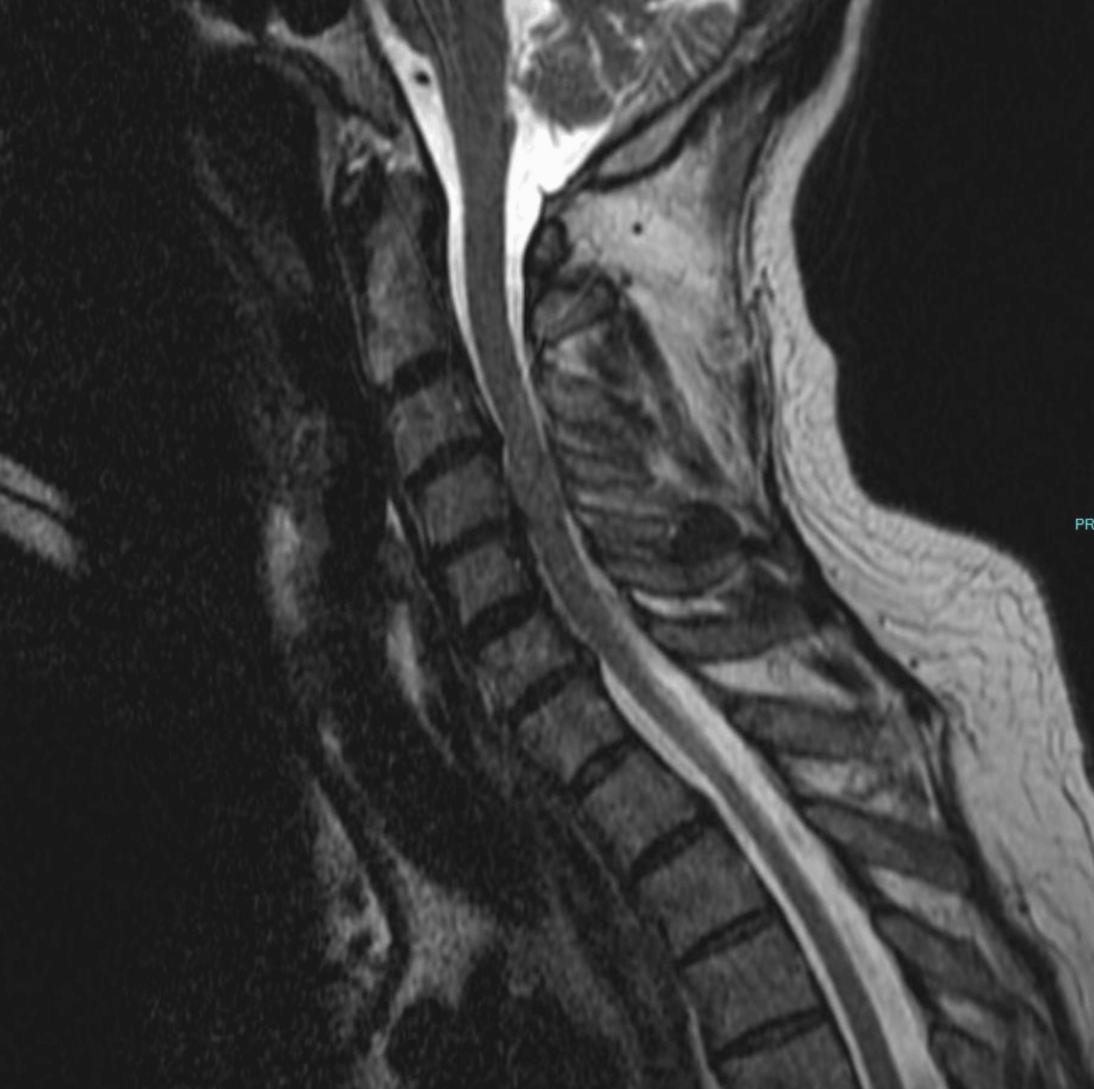
Case E: MRI of cervical spine. There is mild to moderate left neural foraminal disc herniation of the C6-7 level. There is no fracture, spondylolisthesis, or spinal cord mass lesion causing compression.

A 47-year-old man presented to the clinic with unbearable left neck and shoulder pain persisting for almost two months. He attributed the pain to repeated shoveling of snow during the winter. An MRI of the cervical spine revealed some disc herniation and disc space narrowing predominantly at levels C4-7 (Figure 5). His symptoms persisted despite taking NSAIDs and resting. The pain was exacerbated by lying on the affected side, necessitating elevation of his hand at rest. He also experienced numbness in his left upper extremity, particularly after prolonged computer use. The Tafler test was positive, helping to confirm that the patient was suffering from nTOS.

Two weeks of osteopathic therapy in the office had a temporary effect; approximately four months later, the patient experienced a recurrence of symptoms. The same therapeutic maneuver (Tafler test) as described in Case A in combination with OMT (myofascial release, muscle energy, and counterstrain technique) under anesthesia was subsequently performed. The patient was examined four years later and found to have no residual symptoms.

## Discussion

Neurogenic thoracic outlet syndrome (nTOS) is a frequently misdiagnosed pathology, often leading to inappropriate and ineffective treatment approaches. The cases presented in this article highlight the importance of accurate diagnosis and the limitations of current treatments, such as massage therapy or surgery when they are misapplied. Dr. Leonid Tafler’s novel technique of a cervical diagonal hyperflexion stretch when applied to nTOS has demonstrated efficacy in diagnosing the syndrome, providing relief to patients who have not benefited from previous treatment modalities.

Alternative options to detect nTOS are often based on a detailed history and physical examination and various imaging modalities, often leading to multiple differential diagnoses including cervical radiculopathy, rotator cuff injury, or peripheral nerve entrapment [8]. This process can be circumnavigated with the use of Tafler’s method to diagnose nTOS with a specific set of movements. Additionally, there is no large consensus on the best treatment modality for nTOS [5-6]. Most studies on the use of current conservative treatment modalities such as physical therapy show an efficacy that ranges from 27% to 55% [8]. Alternatively, there are surgical options that involve decompression of the brachial plexus with success ranging from 80%-95% [8]. Although effective, this form of treatment is invasive and tends to be a last resort after more conservative measures have been considered. The cervical diagonal hyperflexion technique can be applied as the preliminary method to detect nTOS, avoiding invasive measures. Although not yet used on a large sample of people, there is promise for its effectiveness from the cases presented in this article. After over a decade in practice, Dr. Tafler has used this quick, non-invasive test to diagnose and guide treatment for several patients. The steps to perform the cervical hyperflexion technique are delineated below.

Tafler test

Indication

Diagnosis of neurogenic component of thoracic outlet syndrome (TOS).

Contraindications

Cervical vertebrae fracture, acute trauma, disc herniation, and acute infection.

Procedure

Patient position: Supine.

Physician position: Standing at the head of the treatment table.

**Figure 6:**
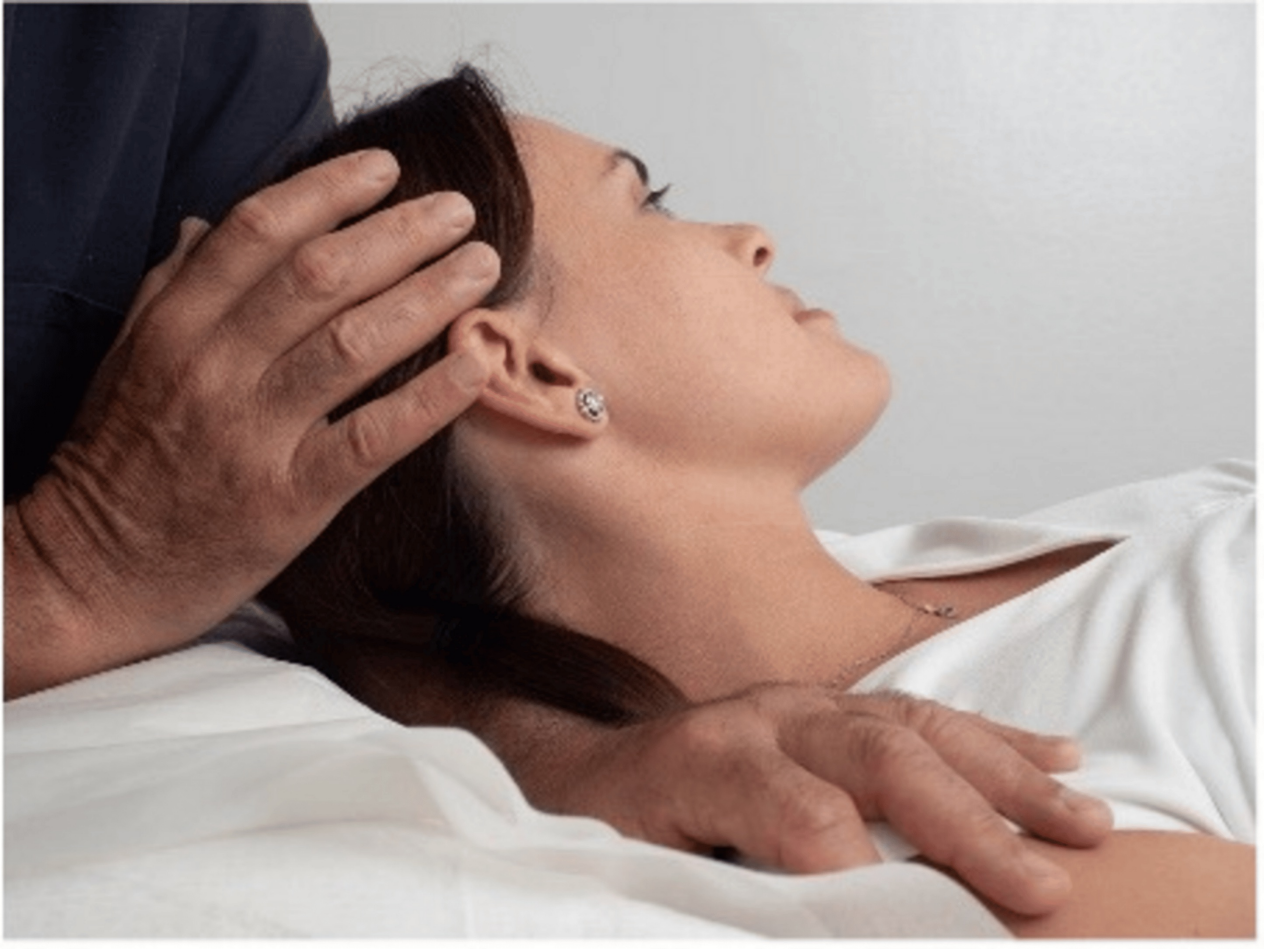
Physician hand positioning. The people depicted in the figure are not real patients but volunteers.

**Figure 7:**
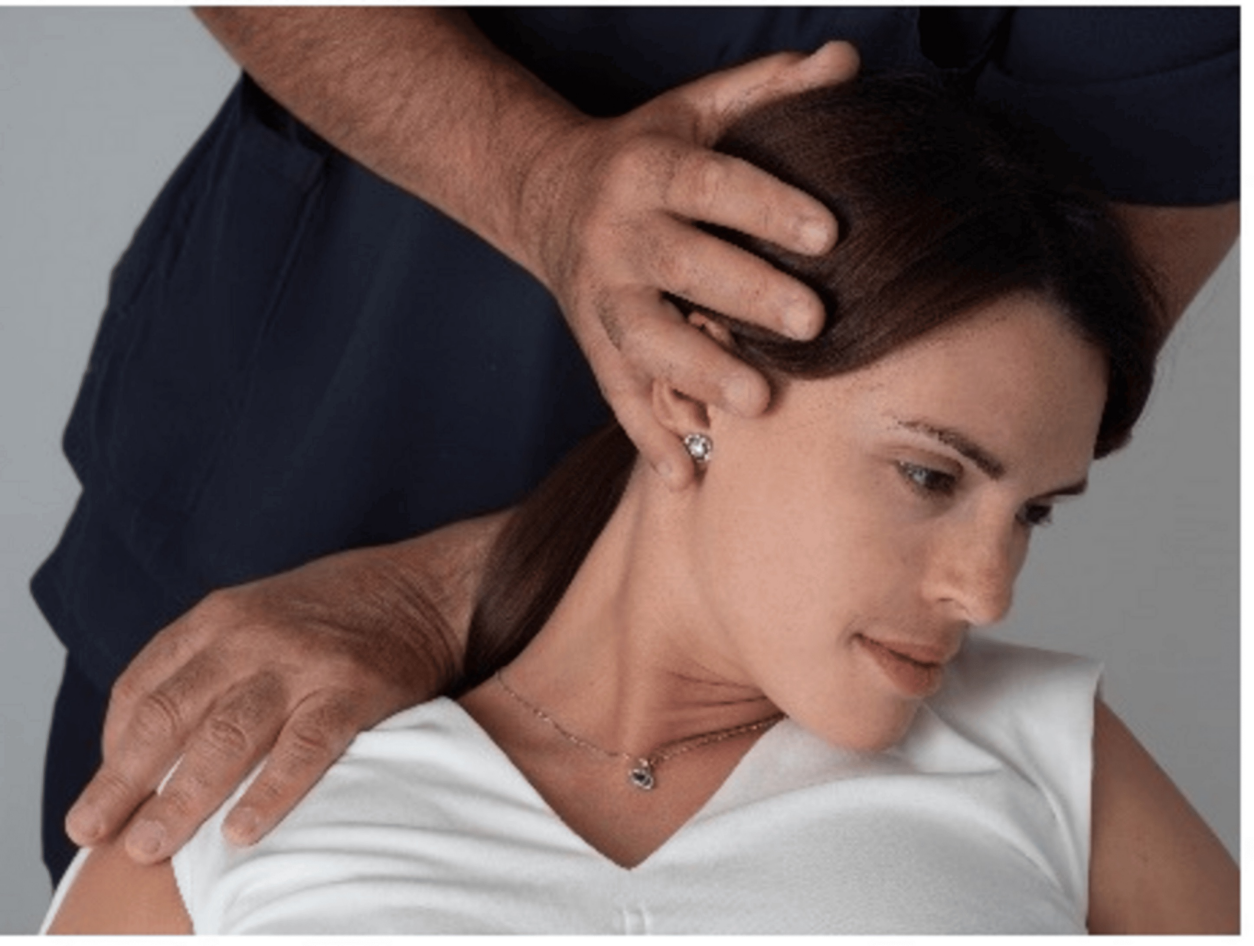
Diagonal hyperflexion. The people depicted in the figure are not real patients but volunteers.

1. The patient’s head is placed on the posterior surface of the examiner’s forearm with the hand placed on the patient’s shoulder on the opposite side, which is the side being tested. The patient’s head should be fully supported on the forearm.

2. The physician slightly flexes, rotates, and side bends the patient’s head away from the dysfunctional side providing the counterforce by pressing on the shoulder, thus gently stretching the soft tissues in the supraclavicular area and relieving the pressure on the brachial plexus (Figures 6, 7).

Assessment: A disappearance of shoulder and arm pain and paresthesia indicates a positive test for neurogenic TOS.

The cases in this article illustrate that there is a need for increased awareness and understanding of nTOS among healthcare providers. By incorporating Dr. Tafler's method into clinical practice, providers can improve diagnostic accuracy and treatment outcomes while avoiding unnecessary or invasive interventions for patients suffering from this condition. Future research should focus on comparing the efficacy of surgical and non-surgical treatments for nTOS to establish evidence-based guidelines for managing the syndrome.

## Conclusions

Our case report introduces an osteopathic test and treatment modality that can benefit patients suffering from thoracic outlet syndrome. In this article, we discussed five cases: one case of neck pain after a workout, two cases of neck/back pain after carrying heavy bags, one case of R arm weakness after a ski accident, and one case of shoulder/neck pain after shoveling snow. The use of this novel osteopathic technique for these patients assisted in diagnosing neurologic manifestations of thoracic outlet syndrome. This newly developed test and treatment modality is important as it can determine the most effective treatment for resolving symptoms and improving patients' activities of daily living. Having an additional diagnostic modality may be essential when facing similar symptoms as described in these cases when there is no resolution with physical therapy and/or surgery. This technique can be performed by any trained osteopathic healthcare professional.
